# *Fusobacterium nucleatum* Promotes the Growth and Metastasis of Colorectal Cancer by Activating E‐Cadherin/Krüppel‐Like Factor 4/Integrin α5 Signaling in a Calcium‐Dependent Manner

**DOI:** 10.1002/mco2.70137

**Published:** 2025-03-10

**Authors:** Xuebing Yan, Xiao Qu, Jiaxin Wang, Ling Lu, Wenjuan Wu, Jingxian Mao, Donglin Li, Ying Wang, Qing Wei, Jianqiang Liu

**Affiliations:** ^1^ Department of Oncology Affiliated Hospital of Yangzhou University Yangzhou China; ^2^ Jiangsu Provincial Innovation and Practice Base for Postdoctor Suining People's Hospital Affiliated Hospital of Xuzhou Medical University Xu Zhou China; ^3^ Department of General Surgery The First Affiliated Hospital of USTC Hefei China; ^4^ Department of Laboratory Diagnostics Changhai Hospital Navy Military Medical University Shanghai China; ^5^ Department of Oncology Northern Jiangsu People's Hospital affiliated to Yangzhou University Yangzhou China; ^6^ Department of Pathology Shanghai Tenth People's Hospital Tongji University Shanghai China; ^7^ Department of Endoscopy Fudan University Shanghai Cancer Center Shanghai China

**Keywords:** calcium signaling, colorectal cancer, *Fusobacterium nucleatum*, integrin α5

## Abstract

Gut microbiota and integrins are known to contribute to colorectal cancer (CRC), but whether they interact has been unclear. Here, we provided evidence that *Fusobacterium nucleatum* upregulated integrin α5 (ITGA5) in CRC in both human patients and murine models. Knocking down *ITGA5* in CRC cells weakened the ability of *F. nucleatum* to stimulate their malignant characteristics. *Fusobacterium nucleatum* increased intracellular Ca^2+^ concentration, which in turn promoted interaction between E‐cadherin and Krüppel‐like factor 4 (KLF4), resulting in KLF4 phosphorylation and translocation in the nucleus, where it induced *ITGA5* transcription and activated the downstream signaling. Knocking down E‐cadherin or chelating Ca^2+^ with BAPTA‐AM antagonized the impact of *F. nucleatum* on KLF4, whereas knocking down *KLF4* or chelating Ca^2+^ antagonized the bacteria's oncogenic role. Knocking down *KLF4* or *ITGA5* attenuated *F. nucleatum–*induced growth of patient‐derived organoids, subcutaneous xenografts, and orthotopic tumors, as well as liver metastasis in nude mice. Integrin α5 antibody antagonized the oncogenic role of *F. nucleatum* in vitro and in vivo. These findings suggest that *F. nucleatum* promotes the growth and metastasis of CRC by activating E‐cadherin/KLF4/integrin α5 signaling in a Ca^2+^‐dependent manner.

## Graphical abstract

Our findings reveal that *Fusobacterium nucleatum* increases intracellular Ca^2+^ concentration, which promotes interaction between E‐cadherin and KLF4, resulting in KLF4 phosphorylation and translocation in the nucleus. The phosphorylated KLF4 induces *ITGA5* transcription and activated FAK/PI3K/Akt1 signaling, finally promoting the growth, invasion, and migration of colorectal cells.

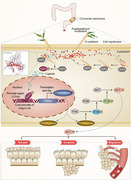

## 1 Introduction

Colorectal cancer (CRC) is the third commonly diagnosed cancer worldwide [1]. Despite the advances in screening and treatments, only 44% of CRC patients are initially diagnosed at an early stage [2]. The development of CRC is a complex biological process involving dysregulation of multiple molecules. For instance, both E‐cadherin and β‐catenin are known to regulate cell adhesion and play a crucial role in the invasion and metastasis of CRC [3]. Low expression of E‐cadherin and high expression of nuclear β‐catenin are associated with poor prognosis [4, 5]. Krüppel‐like factor 4 (KLF4), a transcription factor that regulates tissue homeostasis, has also been implicated in the proliferation, differentiation, and metastasis of CRC cells [6]. KLF4 has also been proposed as a cancer stem cell marker [7].

Accumulating studies have implicated gut microbiota, particularly *Fusobacterium nucleatum*, *Escherichia coli*, and *enterotoxigenic Bacteroides fragilis*, in CRC pathogenesis [8]. In comparison to normal tissues, *F. nucleatum* is significantly enriched in CRC tissues [9]. *Fusobacterium nucleatum* adheres to CRC cells through the adhesion molecule *Fusobacterium nucleatum* adhesin A (FadA) and activates the E‐cadherin/β‐catenin signaling to induce inflammatory and oncogenic responses [10]. Our previous study indicated that *F. nucleatum* promotes CRC development through activating microRNA‐21‐related signaling pathways [11]. We also identified epoxyoctadecenoic acid as an oncogenic metabolite of *F. nucleatum* to drive the epithelial‐mesenchymal transition (EMT) [12]. In addition, *F. nucleatum* was found to regulate the chemosensitivity of CRC cells and could be used as a prognostic biomarker [13]. In contrast to its oncogenic role, a recent study reported that *F. nucleatum* enhances the efficacy of cancer immunotherapy through producing butyric acid [14], adding complexity to this issue.

Integrins have been linked to various hallmarks of cancer [15]. For instance, PRELP could bind with integrin α5 (ITGA5) to activate FAK/AKT signaling pathway, contributing to the growth and metastasis of CRC cells [16]. The primary colorectal tumors secreted integrin beta‐like 1 within extracellular vesicles, which helped promote metastasis by binding to tumor necrosis factor alpha‐induced protein 3 and activated the nuclear factor kappa‐B (NF‐κB) signaling pathway to convert primary fibroblasts into activated fibroblasts [17]. Integrin α2β1 was found to confer resistance to anti‐PD1 therapy by recruiting myeloid‐derived suppressor cells in CRC [18]. Integrin α4β7 inhibited CRC metastasis in a CRC preclinical model by enhancing cancer immunosurveillance [19]. However, whether *F. nucleatum* promotes the initiation and development of CRC by altering integrin expression remains unknown.

We hypothesized that *F. nucleatum* may promote CRC growth and metastasis through regulating integrin‐associated signaling pathways and conducted a series of in vitro and in vivo experiments to test such a hypothesis.

## 2 Results

### 2.1 *F. nucleatum* Upregulates Integrin α5 in CRC

**FIGURE 1:**
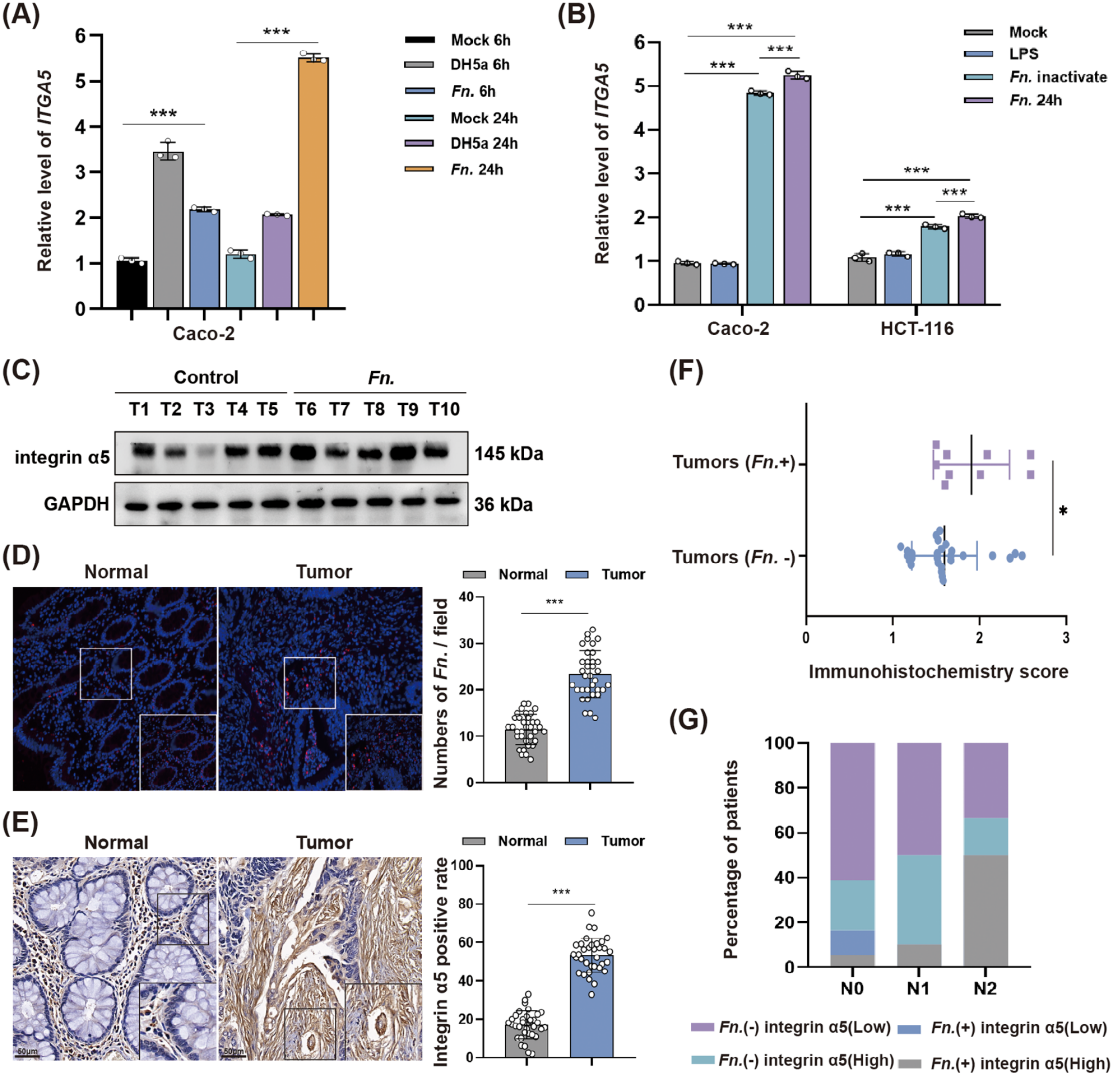
*Fusobacterium nucleatum* upregulates integrin α5 in colorectal cancer. (A) Relative levels of *ITGA5* mRNA encoding integrin α5 in the colorectal cancer (CRC) line Caco‐2 after infection with *E. coli* DH5α or *F. nucleatum* (*Fn*) for 6 or 24 h. Levels were normalized to those of GAPDH. (B) Relative levels of *ITGA5* mRNA in the CRC cell lines Caco‐2 (left) and HCT‐116 (right) after 24‐h treatment with nothing (Mock), lipopolysaccharide (LPS), viable *Fn* or heat‐inactivated *Fn*. (C) Western blot of intestinal tumor lysates from mice in which CRC had been induced using azoxymethane (AOM) and dextran sodium sulfate (DSS), after which the animals were exposed to *Fn* by oral gavage or not (control). Results for five animals in each group are shown. (D) Representative fluorescence micrographs of CRC tissue and adjacent normal tissue from patients after in situ hybridization to detect *Fn* (red). (E) Representative fluorescence micrographs of CRC tissue and adjacent normal tissue from patients after immunohistochemistry to detect integrin α5. (F) Comparison of immunohistochemistry staining scores between patients in cohort 1 whose CRC was infected with *Fn* (*n* = 9) or not (*n* = 25). (G) Distribution of patients in each N stage whose CRC was associated with *Fn* or not and whose tumors expressed low or high levels of integrin α5. *p < 0.05, ***p < 0.001.

Infecting CRC cell lines with *F. nucleatum* for 6 or 24 h upregulated integrin α2, α3, α5, β1, and β3 at the mRNA level (Figure 1A and Figure S1A). Infecting the cells with *Escherichia coli* DH5α for 6 h, but not 24 h, induced similar upregulation in integrin α2 at the mRNA level. The increase in *ITGA5* mRNA was apparent even after the bacteria had been heat‐inactivated (Figure 1B and Figure S1B). However, only the protein expression of integrin α5 was significantly increased after *F. nucleatum* intervention for 6, 24, and 48 h (Figure S1C,D). Therefore, we selected integrin α5 as a target protein of *F. nucleatum* in subsequent experiments.

Administering *F. nucleatum* by oral gavage to mice bearing CRC led to upregulation of integrin α5 protein in tumors (Figure 1C). In patients with CRC (“cohort 1”), tumors showed greater abundance of *F. nucleatum* (Figure 1D) and integrin α5 protein (Figure 1E) than adjacent normal tissue. In comparison to CRC unrelated to the bacteria, the primary tumors associated with *F. nucleatum* had higher expression of integrin α5 protein (Figure 1F). Patients whose primary tumor was associated with *F. nucleatum* and upregulation of integrin α5 protein tended to have more advanced disease (Figure 1G).

### 2.2 *ITGA5* Knockdown Antagonizes the Oncogenic Effects of *F. nucleatum*

**FIGURE 2:**
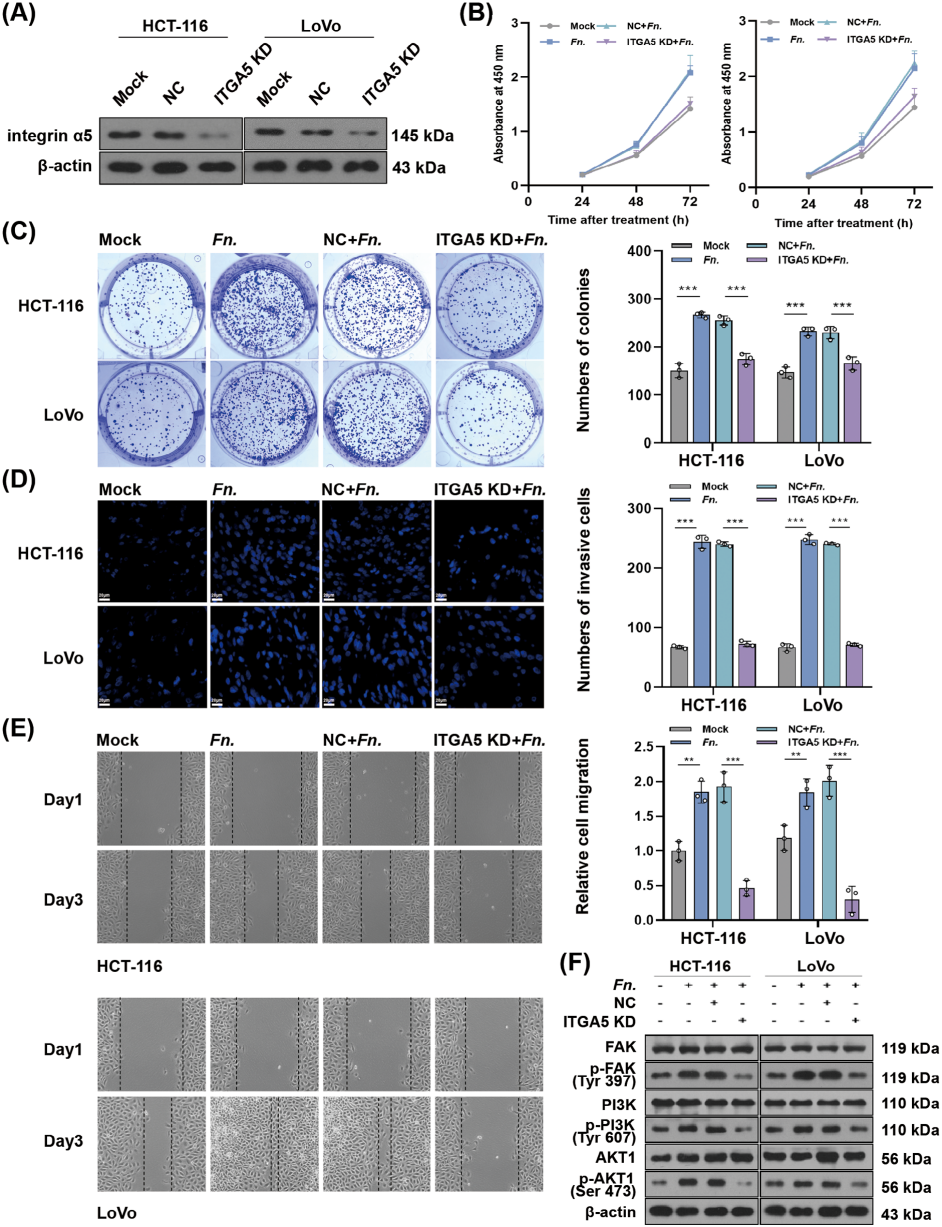
Knockdown of *ITGA5* in vitro antagonizes the oncogenic effects of *F. nucleatum*. (A) Western blot of integrin α5 expression in colorectal cancer (CRC) cells. (B) CCK‐8 assay of proliferation at the indicated times after treatment. (C) Colony formation assay. (D) Transwell assay of invasion ability. (E) Wound healing assay of migration ability. (F) Western blot to assess phosphorylation of FAK, PI3K, and Akt1 in CRC cells. **p < 0.01, ***p < 0.001.

*ITGA5* knockdown in CRC cell lines (Figure 2A) decreased the ability of *F. nucleatum* infection to promote cancer cell proliferation (Figure 2B), colony formation (Figure 2C), invasion (Figure 2D), and migration (Figure 2E). *ITGA5* knockdown also attenuated the ability of *F. nucleatum* to promote phosphorylation of three downstream targets of integrin α5: FAK, PI3K, and Akt1 (Figure 2F).

### 2.3 *F. nucleatum* Upregulates Integrin α5 in CRC by Upregulating KLF4

**FIGURE 3:**
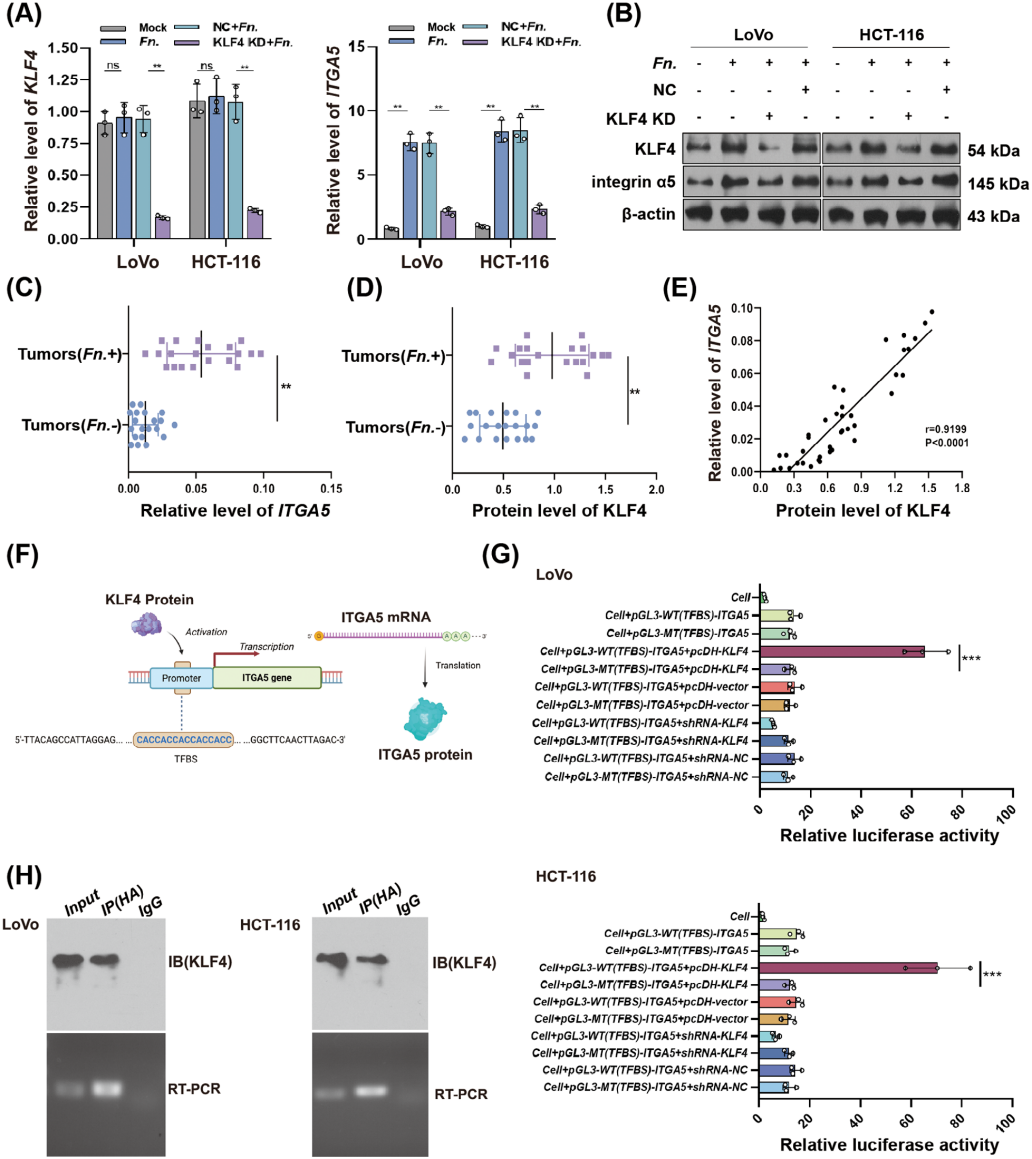
*F. nucleatum* upregulates KLF4 to induce expression of integrin α5. (A) Relative levels of *KLF4* or *ITGA5* mRNA. Levels are normalized to those of β‐actin. Data are from three independent experiments. (B) Western blot of KLF4 or integrin α5 expression in colorectal cancer (CRC) cells. (C and D) Comparison of the mRNA encoding integrin α5 (C) or KLF4 protein (D) in CRC from the 40 patients in cohort 2, stratified by whether their cancer was infected with *Fusobacterium nucleatum* (*Fn*). (E) Correlation between levels of *ITGA5* mRNA and levels of KLF4 protein in CRC. The correlation parameters were determined using Spearman's rank correlation test. (F) Predicted binding site for KLF4 in the promoter of the *ITGA5* gene encoding integrin α5. (G) A luciferase reporter assay detected the transcriptional activity of *ITGA5* promoter containing wild‐type (WT) or mutated (MT) sequences at the putative binding site of KLF4. (H) Chromatin immunoprecipitation (ChIP) assay in HCT‐116 and LoVo cells. **p < 0.01, ***p < 0.001.

This set of experiments focused on the transcription factor KLF4 since it has been shown to regulate integrin expression [20, 21, 22]. Infecting CRC cell lines with the bacteria upregulated KLF4 at the protein but not mRNA level (Figure 3A,B). *KLF4* knockdown reversed the bacteria induced upregulation of integrin α5 protein and significantly reduced its mRNA level. Consistent with these results, primary human CRC associated with *F. nucleatum* (cohort 2, Table S2) had higher levels of *ITGA5* mRNA and KLF4 protein than CRC samples unrelated to *F. nucleatum* (Figure 3C,D). Across all patients in cohort 2, levels of mRNA encoding integrin α5 correlated positively with levels of KLF4 protein (Figure 3E). The correlation analysis revealed the *F. nucleatum* abundance, in which ITGA5 and KLF4 levels were significantly correlated with TNM stage, but unrelated with other clinical features.

The bioinformatics analysis uncovered the potential binding site of KLF4 to the promoter region of the gene encoding integrin α5 (Figure 3F). In a dual‐luciferase reporter assay, the transcriptional activity of wild‐type ITGA5 promoter but not its mutant in CRC cells was increased by KLF4 overexpression and decreased by KLF4 knockdown (Figure 3G). In chromatin immunoprecipitation (ChIP) assay, DNA fragments of the *ITGA5* promoter containing the TFBS were detected in the eluates of KLF4 protein purified by immunoprecipitation in CRC cells, confirming that the KLF4 protein can bind to the *ITGA5* promoter (Figure 3H). The above findings were also confirmed using 293T cells (Figure S2A,B). A bandshift assay revealed KLF4 could bind with the specific DNA probe for *ITGA5* promoter instead of the mutated DNA probe, further confirming the binding of KLF4 on the TFBS of *ITGA5* promoter (Figure S2C).

### 2.4 *F. nucleatum* Promotes KLF4 Translocation to the Nucleus Through a Pathway Involving E‐Cadherin and Ca^2+^

**FIGURE 4:**
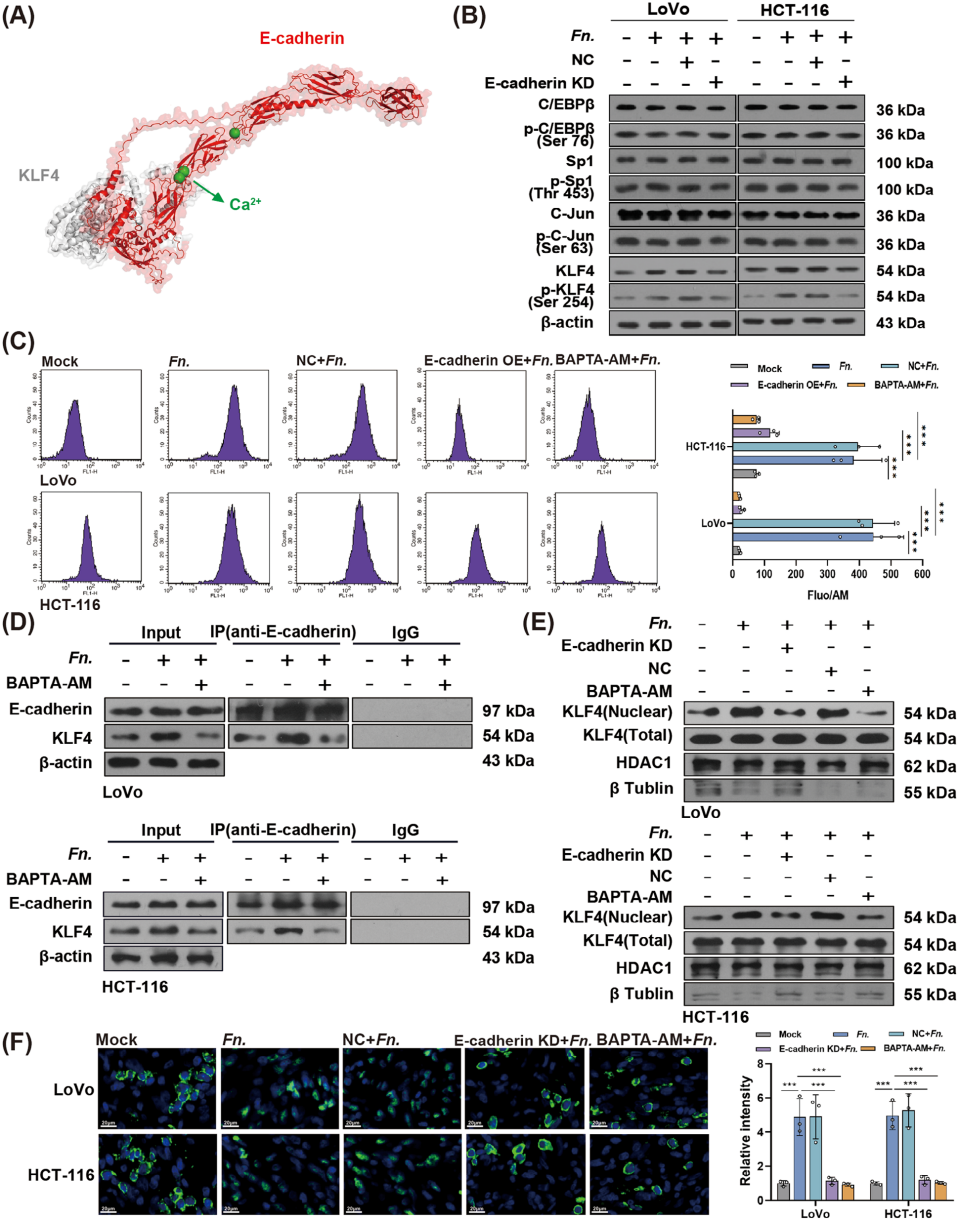
*F. nucleatum* promotes translocation of KLF4 to the nucleus through a process dependent on E‐cadherin and Ca^2+^. (A) Molecular docking model of phosphorylated KLF4 (Ser‐254), E‐cadherin, and Ca^2+^. (B) Western blot of expressions of transcription factors known to induce expression of integrin α5 in *Fusobacterium nucleatum* (*Fn*)–infected colorectal cancer (CRC) cells with or without E‐cadherin knockdown. (C) Flow cytometry to assess intracellular Ca^2+^ concentrations in CRC cells overexpressing E‐cadherin (OE) or treated with the Ca^2+^ chelator BAPTA‐AM. (D) Co‐immunoprecipitation to detect binding between KLF4 and E‐cadherin. (E) Western blot for detecting the protein expression of nuclear KLF4 in *Fn*‐infected CRC cells with E‐cadherin knockdown or BAPTA‐AM treatment. (F) Representative fluorescence micrographs of cells treated in the indicated ways, and then immunostained against KLF4 (green) to determine its subcellular localization. Nuclei were counterstained with DAPI (blue). ***p < 0.001.

KLF4 must first be phosphorylated and translocate to the nucleus to produce biological function [23]. We next examined whether *F. nucleatum* affects these processes. Considering a crucial role of E‐cadherin in *F. nucleatum* infection [10, 24, 25], we constructed three molecular docking models: model 1: KLF4 and E‐cadherin (Figure S3A); model 2: phosphorylated KLF4 (Ser‐254) and E‐cadherin (Figure S3B); model 3: phosphorylated KLF4 (Ser‐254), E‐cadherin, and Ca^2+^ (Figure 4A). The binding energy was highest in model 3 (−58.6 kcal/mol), followed by −52.2 kcal/mol in model 2 and −50.1 kcal/mol in model 1. The GST pull‐down assay verified direct interaction between E‐cadherin and KLF4 (Figure S3C). Knocking down E‐cadherin in CRC cells partially reversed the ability of *F. nucleatum* to induce phosphorylation of KLF4, without affecting the phosphorylation of the transcription factors C/EBPβ, Sp‐1 or c‐Jun, which are also known to turn on expression of integrin α5 [26, 27] (Figure 4B). Given the importance of Ca^2+^ for E‐cadherin function [28], we next examined whether Ca^2+^ is required for *F. nucleatum* to upregulate KLF4. The results indeed showed significantly increased intracellular Ca^2+^ in CRC cells upon *F. nucleatum* infection (Figure 4C), with greater interaction between E‐cadherin and KLF4 (Figure 4D). Chelating Ca^2+^ with BAPTA‐AM reduced the interaction between E‐cadherin and KLF4. Finally, *F. nucleatum* infection increased the translocation of KLF4 into the nucleus, which was partially reversed by E‐cadherin knockdown or BAPTA‐AM (Figure 4E,F).

### 2.5 KLF4 Knockdown or Ca^2+^ Chelation in Vitro Antagonizes the Oncogenic Effects of *F. nucleatum*

The experiments above suggested that *F. nucleatum* drives onset and progression of CRC by upregulating KLF4 followed by integrin α5 via a Ca^2+^‐dependent process. This implies that downregulating KLF4 or chelating Ca^2+^ with BAPTA‐AM should weaken its oncogenic effects. Indeed, either treatment partially reversed the ability of *F. nucleatum* to promote cancer cell proliferation, colony formation, invasion, and migration in vitro (Figure S4).

### 2.6 *KLF4* and *ITGA5* Knockdown in Vivo Antagonizes the Oncogenic Effects of *F. nucleatum*

*KLF4* or *ITGA5* knockdown inhibited CRC growth (Figure S5A–C) and decreased Ki67 in mice carrying subcutaneous HCT‐116 xenografts (Figure S5D,E). The opposing effects of *F. nucleatum* infection and *KLF4*/*ITGA5* knockdown were mirrored in the levels of phosphorylation of FAK, PI3K, and Akt1 in tumors (Figure S5F).

**FIGURE 5:**
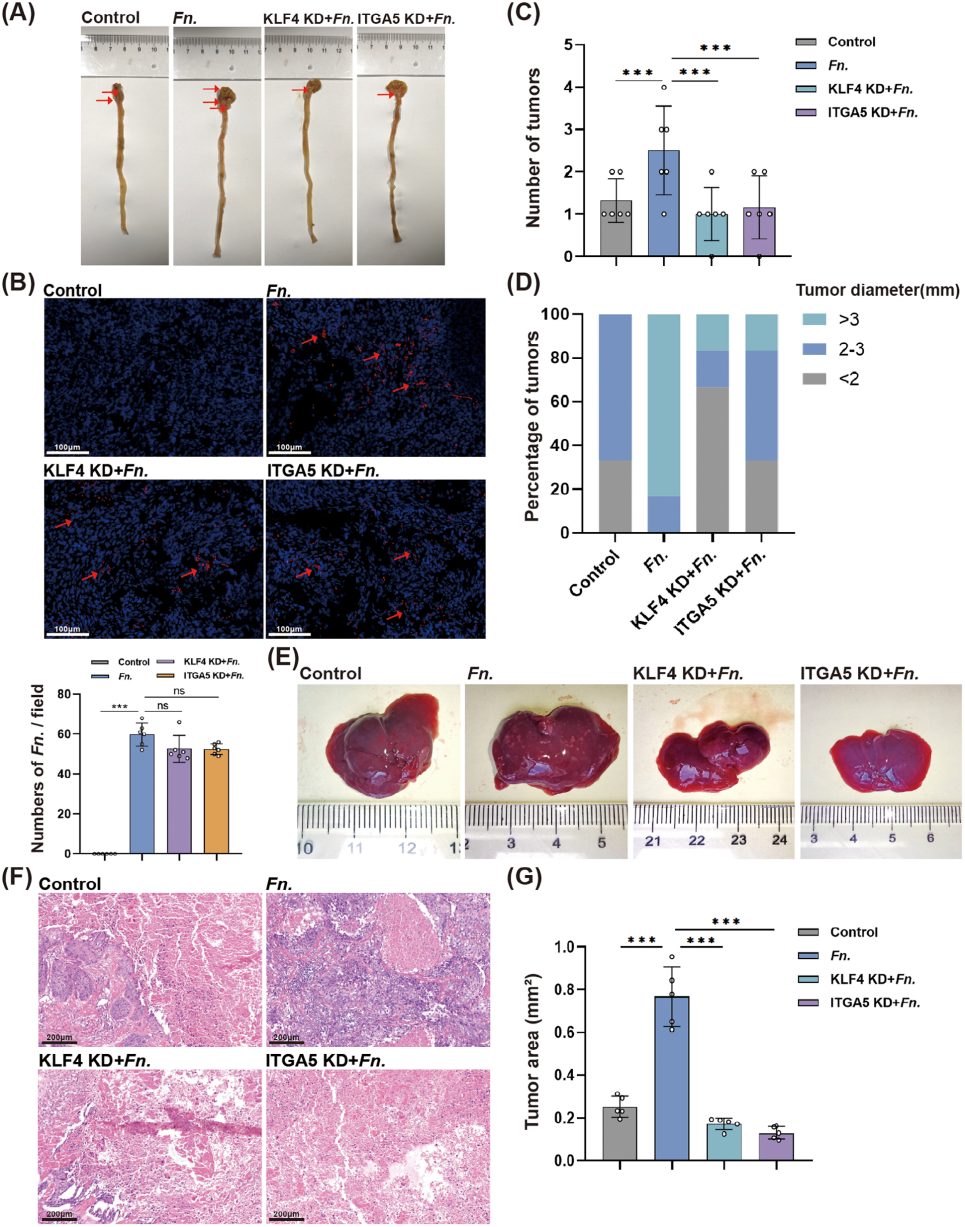
Knockdown of *KLF4* or *ITGA5* in vivo antagonizes the oncogenic effects of *F. nucleatum*. (A) Representative photographs of intestines. Red arrows indicate intestine tumors. (B) Representative fluorescence micrographs of intestinal tumors subjected to in situ hybridization to detect *Fusobacterium nucleatum* (red). Red arrows indicate clusters of bacteria. (C) Numbers of intestinal tumors. (D) Distribution of tumor diameters. (E) Representative photographs of livers. (F) Representative micrographs of liver sections after staining with hematoxylin‐eosin. (G) Comparison of metastatic tumor area in liver. ***p < 0.001.

In mice carrying orthotopic CRC (Figure 5A,B), *F. nucleatum* infection significantly increased the number and diameter of tumors in the intestine; such effect was partially attenuated by knockdown of *KLF4* or *ITGA5* (Figure 5C,D). *Fusobacterium nucleatum* infection also increased the rate of metastasis of CRC cells to the liver; such effect was also attenuated by knockdown of *KLF4* or *ITGA5* (Figure 5E–G).

### 2.7 *KLF4* and *ITGA5* Knockdown in Patient‐Derived Organoids Antagonizes the Oncogenic Effects of *F. nucleatum*

**FIGURE 6:**
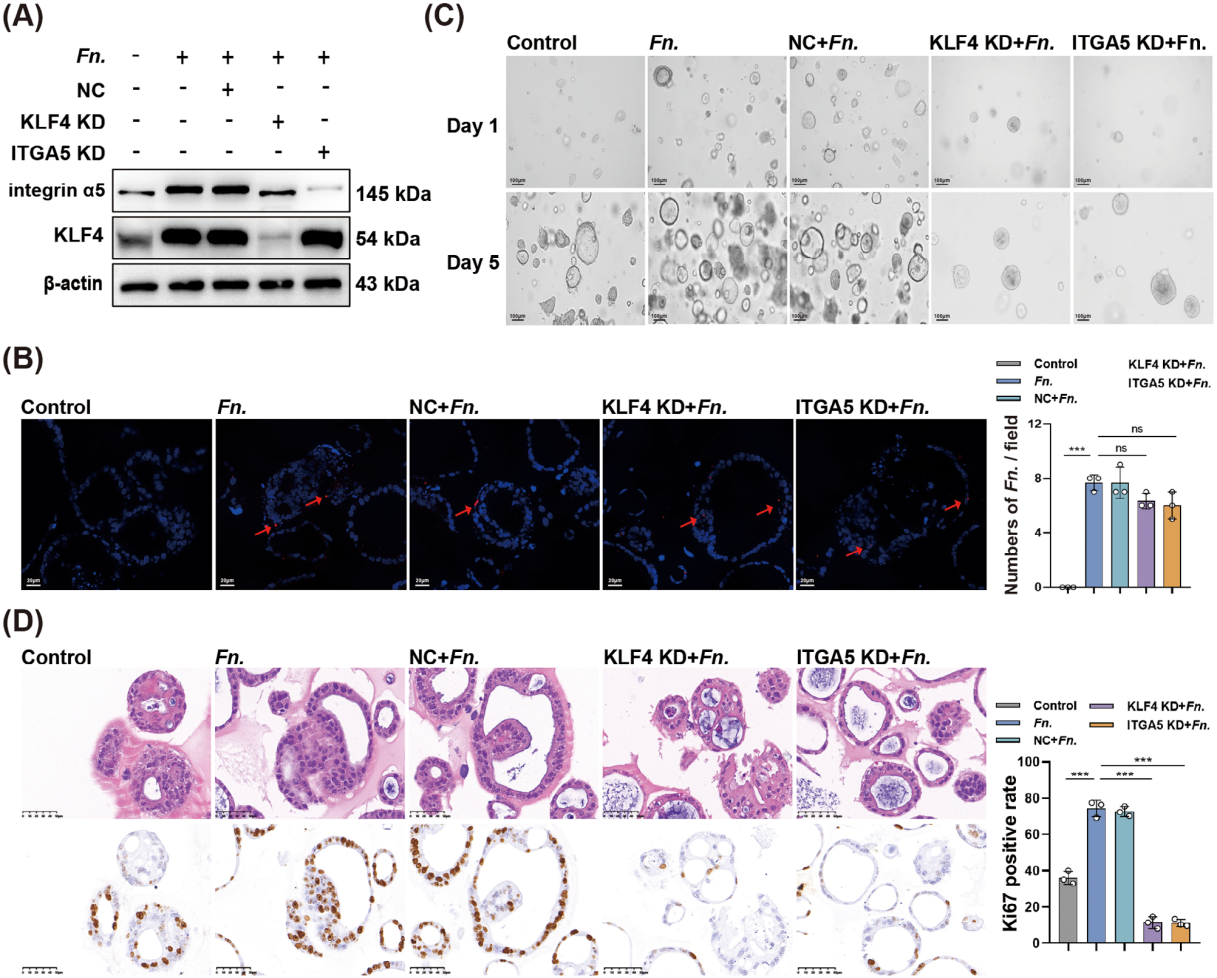
Knockdown of *KLF4* or *ITGA5* in colorectal cancer organoids antagonizes the oncogenic effects of *F. nucleatum*. (A) Validation of *KLF4* or *ITGA5* knockdown in *F. nucleatum* infected organoids. (B) Representative fluorescence micrographs of organoids after in situ hybridization to detect *F. nucleatum* (red). Red arrows indicate clusters of bacteria within organoids. (C) Representative micrographs of organoids. (D) Representative micrographs of organoids after staining with hematoxylin‐eosin (upper row) or immunostaining against Ki67 (lower row). ***p < 0.001.

In patient‐derived CRC organoids (Figure 6A), knocking down *KLF4* or *ITGA5* partially reversed the ability of *F. nucleatum* infection to promote organoid growth (Figure 6B,C) and to increase Ki67 (Figure 6D).

### 2.8 Integrin a5 Antibody Antagonizes the Oncogenic Effects of *F. nucleatum* in Vitro and in Vivo

Treatment of cultured CRC cells with an integrin α5 antibody attenuated the effects of *F. nucleatum* infection on cell proliferation (Figure S6A), colony formation (Figure S6B), invasion (Figure S6C), and migration (Figure S6D). In mice carrying subcutaneous HCT‐116 xenografts, integrin α5 antibody treatment inhibited the growth (Figure S6E–G) and decreased Ki67 (Figure S6H,I) in *F. nucleatum*–infected xenografts.

## 3 Discussion

**FIGURE 7:**
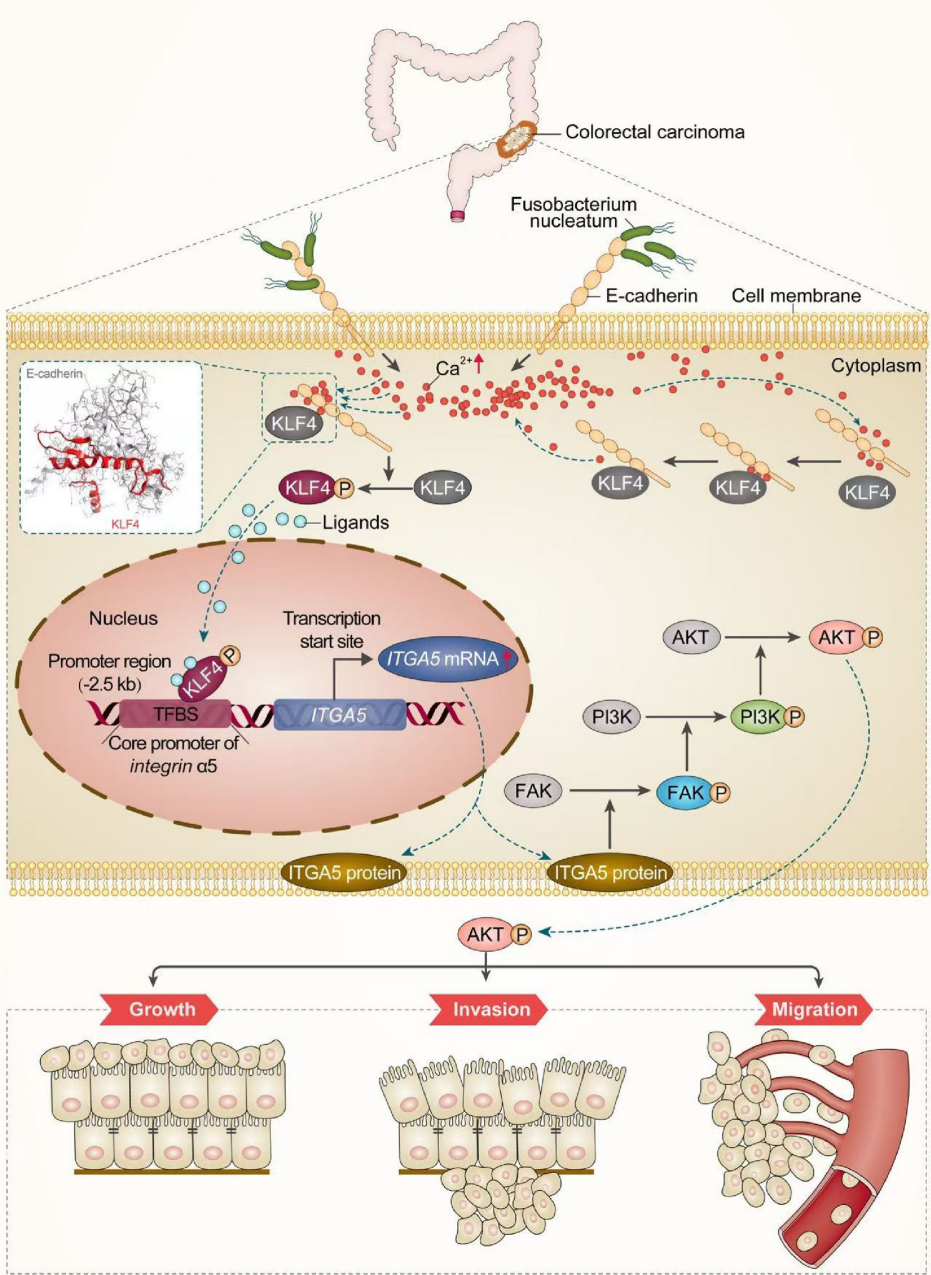
Proposed scheme in which *F. nucleatum* promotes colorectal cancer development by activating KLF4/ITGA5 signaling through a mechanism involving E‐cadherin and Ca^2+^.

Results from the current study indicate *F. nucleatum* promotes the progression of CRC by upregulating the transcription factor KLF4, promoting its phosphorylation and translocation to the nucleus via a mechanism dependent on E‐cadherin and Ca^2+^. Consistent with the oncogenic role of integrins [29, 30], KLF4 turns on integrin α5 to activate signaling involving FAK, PI3K, and Akt1 (Figure 7). Our findings not only clarify how gut microbiota can promote gastrointestinal cancer, but they also identify several potential therapeutic targets, including KLF4, integrin α5, and calcium channels.

The current study may help explain why the presence of *F. nucleatum* in colorectal tumors can influence their response to anticancer therapies [31]. Indeed, the abundance of certain gut microbiota has been linked to treatment response and prognosis in CRC as well as other cancers [32, 33]. In addition to secreting oncogenic metabolites [12, 34], *F. nucleatum* may also exert oncogenic effects by directly activating signaling pathways that promote cancer transformation and metastasis in CRC [35, 36]. Another gut microbe, *E. coli*, can promote CRC metastasis by permeabilizing the gut endothelium, facilitating the passage of tumor cells into the circulation [37]. Among all integrins that we examined, only integrin α5 was upregulated by *F. nucleatum* in CRC cells. Previous work has reported integrin α5 upregulation in CRC and its association with poor survival outcomes [38, 39]. Integrin β4 has been shown to promote metastasis to the lungs [40], whereas integrin β1 promotes metastasis to the liver [41]. Future work should examine the roles of different integrins in promoting CRC in different contexts.

The current study extended the list of cancers involving oncogenic roles of KLF4 from lung, breast, and prostate cancers to CRC [23]. *Fusobacterium nucleatum* infection of CRC cells increased KLF4 at the protein but not mRNA level, implying post‐transcriptional regulation, most likely phosphorylation at Ser254 and possibly other sites [23]. Alternative mechanisms include methylation and acetylation. For instance, arginine N‐methyltransferase 5 (PRMT5) interacts with KLF4 and induces the methylation of arginine 374, 376, and 377 in KLF4, contributing to KLF4 transcriptional activity [42]. KLF4 interacts with and is acetylated by p300/CREB‐binding protein at lysine 225 or 229, which inhibits the ability of KLF4 to activate downstream targets [43]. Previous studies have suggested that the opposing roles of KLF4 in cancer cells (suppressing vs. promoting) may derive from its subcellular localization [23]. For example, poor outcome in non‐small cell lung cancer patients has been associated with high expression of KLF4 in the nucleus but low KLF4 expression in the cytoplasm [44]. Nuclear localization of KLF4 contributes to malignant transformation of epithelial cells and is correlated with poor outcome in patients with early‐stage infiltrating ductal carcinoma [45]. In CRC, KLF4 can act as a tumor suppressor through inducing apoptosis and inactivating WNT pathway in the early stage, but also as an oncogene through inducing stemness in the late stage, suggesting its context‐dependent role [6]. The current study showed phosphorylated KLF4 in CRC cells upon *F. nucleatum* infection translocated into cell nucleus to activate *ITGA5* transcription and its related oncogenic signaling pathway. These findings suggested that *F. nucleatum–*induced nuclear localization of KLF4 may be a crucial driver switching its role from a tumor suppressor to an oncogene in CRC.

E‐cadherin is a membrane glycoprotein that regulates cellular adhesion and its loss is correlated with aggressive phenotype of numerous epithelial cancers [46]. Previous studies have identified E‐cadherin as a crucial signal molecule of *F. nucleatum*–induced biological functions. *Fusobacterium nucleatum* activates oncogenic β‐catenin signaling through the binding of FadA adhesin with E‐cadherin [10]. *Fusobacterium nucleatum* induces colon anastomosis through upregulating matrix metalloproteinase 9 expression, which is dependent on E‐cadherin/β‐catenin signaling pathway [25]. The current study demonstrated a novel role of E‐cadherin in *F. nucleatum*–induced CRC development through its interaction with KLF4. E‐cadherin is encoded by the *CDH1* gene, which contains four E‐box regions that can be readily recognized by transcription factors [47]. We also found *F. nucleatum* treatment increases the intracellular Ca^2+^ level, which is crucial for the interaction between E‐cadherin and KLF4. Increased intracellular Ca^2+^ level has been closely associated with proliferation, invasion, and migration of CRC cells [48]. Increased Ca^2+^ flux in mucosal epithelial cells has been shown in buccal epithelial cells upon contact with *E. coli* [49]. Functional assays in the current study revealed decreased oncogenic effects of *F. nucleatum* upon Ca^2+^ chelation, suggesting intracellular Ca^2+^ signaling may be a potential therapeutic target for *F. nucleatum*–infected CRC patients.

The current study has several limitations. First, the prognostic significance of *F. nucleatum* infection in CRC patients was not examined due to the relatively small sample size. Whether detection of *F. nucleatum* and ITGA5 can be used as a prognostic tool requires future studies. Second, only FAK/PI3K/Akt1 signaling pathway was examined. Third, integrin α5 antibody was used for drug intervention experiments in vitro and in vivo, but related safety assessment is lacking and should be investigated in future studies.

## 4 Conclusion

*F. nucleatum* increases intracellular Ca^2+^, which in turn activates a pathway involving E‐cadherin, KLF4, and integrin α5 to drive progression of CRC. These findings provided an example of how gut microbes can contribute to cancer and identified several potential biomarkers for CRC.

## 5 Materials and Reagents

### 5.1 Patient Information

This study was approved by the Institutional Review Board of the Affiliated Hospital of Yangzhou University and carried out according to the Declaration of Helsinki. A consecutive series of 34 patients with CRC was recruited at our hospital between January 2019 and December 2022 (cohort 1), while another series of 40 patients was recruited between January 2019 and June 2020 (cohort 2) (Table S1). Surgical biopsies of tumors and adjacent normal tissue were taken from cohort 1, embedded in paraffin, and analyzed using quantitative PCR, fluorescence in situ hybridization (FISH), and immunohistochemistry. Biopsies were taken from cohort 2 and processed fresh in quantitative PCR and enzyme‐linked immunosorbent assays. All participants signed written informed consent on enrollment.

### 5.2 Construction of Expression Plasmids and Recombinant Lentivirus

Plasmids encoding short hairpin RNAs for knockdown experiments (Table S3) were constructed and verified by DNA sequencing. Recombinant lentivirus was generated by co‐transfecting 293T cells with recombinant vectors (2 µg each) and 10 µg of the pPACK Packaging Plasmid Mix (System Biosciences, USA) using X‐tremeGENE 9 DNA Transfection Reagent (Roche, Switzerland). After 48‐h incubation, lentivirus in the culture medium was harvested and used to infect CRC cells. Clones that had stably integrated the transgenic sequences were selected using puromycin.

### 5.3 Quantitative Real‐Time PCR

Total RNA was extracted from tissues or cells using TRIzol (Thermo Fisher Scientific, USA), and then reverse‐transcribed into cDNA using M‐MLV reverse transcriptase (Promega, USA). The cDNA served as template in quantitative PCR using the SYBR Green PCR Kit (Takara Biotechnology, Japan). Primers are shown in Table S3. Levels of target mRNAs were quantified using the 2^−ΔΔT^ method and normalized to those of internal control mRNA encoding either GAPDH or β‐actin.

### 5.4 Quantitation of *F. nucleatum*

The abundance of *F. nucleatum* was quantitated as the number of copies of the bacterial gene encoding 16S rRNA. Genomic DNA was extracted from fresh tissues using the QIAamp DNA Mini Kit (Qiagen, Germany), or from paraffin‐embedded tissues using the QIAamp DNA FFPE Tissue Kit (Qiagen, Germany). Quantitative PCR was performed using the SYBR Premix Ex Taq (Takara Biotechnology, Japan) and primers in Table S3 on a thermal cycling system (Thermo Fisher Scientific, USA). The reaction conditions were as follows: 10 min at 95°C for initial denaturation, followed by 40 cycles of 1 min at 95°C for denaturation, 20 s at 60°C for primer annealing, and 60 s at 56°C for primer extension. Levels of 16S rRNA were determined using the 2^−ΔT^ method relative to levels of the internal control mRNA encoding SLCO2A1.

In some experiments, abundance of *F. nucleatum* was quantitated based on FISH using a *Fusobacterium* 16S rRNA‐directed probe. Paraffin‐embedded tissues were dewaxed in xylene, rehydrated in alcohol, incubated with antigen retrieval solution containing proteinase K, and then incubated with cyanine3‐labeled probe (5’‐CGCAATACAGAGTTGAGCCCTGC‐3’; Sangon Biotech, China). Nuclei in samples were counterstained using 4,6‐diamino‐2‐phenyl indole (DAPI, Sangon Biotech, China) and examined under a fluorescence microscope (Olympus Corporation, Japan). The images were analyzed using Image Pro Plus 7.0 software.

### 5.5 Western Blotting

Total protein was extracted from cells or tissues using M‐PER mammalian protein extraction reagent (Thermo Fisher Scientific, USA), fractionated on a 10% SDS–polyacrylamide gel, and transferred to a polyvinylidene difluoride membrane. Membranes were blocked with 5% skim milk, incubated overnight at 4°C with a primary antibody, and then with an appropriate secondary antibody for 1.5 h at room temperature (Table S4). Antibody binding was detected using a chemiluminescence detection kit (Thermo Fisher Scientific, USA). Levels of target proteins were normalized to levels of GAPDH, β‐actin, or HDAC1.

### 5.6 Immunohistochemistry

Paraffin‐embedded tissues were cut into 5‐µm sections, dewaxed in xylene, rehydrated in alcohol and heated in a microwave oven to retrieve antigens. Endogenous peroxidase activity was blocked using 0.3% hydrogen peroxide, then sections were washed three times with phosphate‐buffered saline (PBS) and incubated overnight at 4°C with a primary antibody followed by incubation with an appropriate secondary antibody for 1 h (Table S4). Antibody binding was detected using diaminobenzidine tetrahydrochloride (Thermo Fisher Scientific, USA).

### 5.7 Detection of KLF4 Protein

Levels of KLF4 in patient CRC tissues were quantified using a commercial enzyme‐linked immunosorbent assay (Abcam, UK). Absorbance was measured at 450 nm.

Thin sections of CRC cells were fixed on slides with 4% paraformaldehyde, incubated with PBS containing 0.2% Triton, and blocked with bovine serum albumin for 1 h. Sections were incubated overnight at 4°C with primary antibody, washed three times in PBS, then incubated in secondary antibody for 1 h (Table S4) before visualization using DAPI under a fluorescence microscope.

### 5.8 Cell Culture

Human CRC cell lines Caco‐2, HCT‐116, and LoVo, as well as the human kidney cell line 293T and *F. nucleatum* strain ATCC 25586 were purchased from the American Type Culture Collection (Manassas, USA). The cell lines Caco‐2 and HCT‐116 were isolated from the primary tumors of CRC patients; LoVo cell line was isolated from a metastatic tumor nodule of an adult male CRC patient. We selected the cell lines HCT‐116 and LoVo for the functional assays according to our previous studies [11, 12]. *Escherichia coli* DH5α was purchased from Thermo Fisher Scientific. Cells were cultured at 37°C in an atmosphere of 5% CO_2_, as previously described [11, 12], and cell lines were verified using short‐tandem repeat analysis and tested for Mycoplasma. In assays in which cell lines were infected with *F. nucleatum*, the multiplicity of infection was 1000:1. For antibody treatment, cells were inoculated with a medium containing anti‐integrin α5 antibody (1 ng/mL) for 24 h.

### 5.9 Cell Proliferation Assay

Cell proliferation was examined after 4‐h culture using a CCK‐8 assay (Solarbio Life Sciences, China) at 450 nm using a microplate reader (Thermo Fisher Scientific, USA).

### 5.10 Colony Formation Assay

Cells were seeded into six‐well culture plates, cultured for 2 weeks, fixed with 4% paraformaldehyde, stained with crystal violet, and counted under a microscope [50].

### 5.11 Transwell Assay

For invasion assay, Matrigel (Corning, USA) was added to the upper chamber of transwell dishes (Thermo Fisher Scientific, USA), and incubated for 1 h at 37°C. Cells that had been suspended in serum‐free medium were seeded into the upper chamber, then medium containing 10% fetal bovine serum was added to the lower chamber. Then, the membrane was cut off to evaluate the number of DAPI stained cells passing through the membrane.

### 5.12 Wound Healing Assay

Cells were cultured in six‐well plates until confluence. The medium was removed, and a sterile pipette tip was dragged across the monolayer. Cells were then incubated in serum‐free medium for various time prior to photography to determine the scratch width.

### 5.13 Fluorescence‐Based Detection of Intracellular Ca^2+^

Cells were suspended in PBS containing 5 µM fluo‐3 AM (Sangon Biotech, China) for 1 h at 37°C, washed three times in staining buffer (Thermo Fisher Scientific, USA), suspended in staining buffer, and analyzed by flow cytometry with an excitation wavelength of 488 nm and an emission wavelength of 525 nm.

### 5.14 Simulated Docking Between KLF4 and E‐Cadherin

UniProt (Universal Protein) is a protein database containing protein sequence, functional information, and index to research papers, which integrates three major databases including EBI (European Bioinformatics Institute), SIB (the Swiss Institute of Bioinformatics), and PIR (Protein Information Resource). With KLF4 and E‐cadherin as keywords, the protein structures were retrieved from UniProt, and the human species were screened in the database. KLF4_HUMAN (UniProt ID: O43474) and CADH1_HUMAN (UniProt ID: P12830) were selected. The 3D structure modelings were performed using the Alphafold3. Model 1: The full‐length sequences of protein KLF4 and protein E‐cadherin were used to construct the protein complex model 1. Model 2: The full‐length sequences of protein KLF4 and protein E‐cadherin, and phosphorylated amino acid site Ser‐254 of protein KLF4 were used to construct the protein complex model 2. Model 3: The full‐length sequences of protein KLF4 and protein E‐cadherin, phosphorylated amino acid site Ser‐254 of protein KLF4, and three Ca^2+^ ions (three Ca^2+^ ions are usually bound at the interface of each cadherin domain and strengthen the connections, imparting a strong curvature to the full‐length ectodomain) were used to construct the protein complex model 3.

### 5.15 Co‐Immunoprecipitation to Detect Binding Between KLF4 and E‐Cadherin

Cells were lysed in non‐denaturing lysis buffer containing protease inhibitors, and the lysate was incubated with anti‐KLF4 antibody linked to protein A/G sepharose beads (Abcam, UK). Immunocomplexes were precipitated using a commercial kit (Abcam, UK). Precipitated beads were washed using 0.1% bovine serum albumin in PBS, then boiled in protein loading buffer for 5 min. The eluted protein was analyzed by western blotting.

### 5.16 Chromatin Immunoprecipitation to Detect Binding Between KLF4 and the Integrin α5 Promoter

Cells were cross‐linked, lysed, and disrupted with ultrasound using a chromatin immunoprecipitation assay kit (Abcam, UK). The lysate was incubated overnight with primary antibody against KLF4 or IgG (Table S4), then protein A‐agarose (Abcam, UK) was added, and the beads were precipitated. Immunoprecipitated chromatin was eluted and analyzed for the presence of *ITGA5* promoter using the primers in Table S3.

### 5.17 Dual‐Luciferase Reporter Assay

The promoter location in the chromosome was analyzed using the NCBI database. A 2.0‐kb region of DNA upstream of the transcription start site of the human *ITGA5* gene, as predicted to be the *ITGA5* promoter by Promoter 2.0, was amplified using PCR with the primers in Table S3. The promoter region was subcloned into the pGL3 vector (Promega, USA) to derive pGL3‐WT(TFBS)‐ITGA5 that carries the wild‐type (WT) sequence (5′‐CACCACCACCACCACC‐3′) for the putative TFBS. The binding site was predicted using JASPAR. The primer sequence for the mutant to derive pGL3‐MT(TFBS)‐ITGA5 are as follows: 5′‐ACCACCCCACCCAACC‐3′. The coding region of KLF4 was amplified from the human cDNA as the template to construct pcDNA‐KLF4.

### 5.18 Electrophoretic Mobility Shift Assay

Purified KLF4 (0.6 µg) was mixed with a radiolabeled probe (0.1 pmol) (Sangon Biotech, China) containing wild‐type or mutant sequences in its predicted binding site within the *ITGA5* promoter (Table S3) and incubated for 30 min at 4°C. Binding was assessed using the Light Shift Chemiluminescent EMSA Kit (Thermo Fisher Scientific, USA) as previously described [51].

### 5.19 GST Pull‐Down Assay

The transformed *E. coli* BL21 (DE3) cells (Beyotime, China) carrying the pET‐GST‐E‐cadherin or pET‐GST (Novagen, Germany) were cultured in LB medium containing ampicillin. Expression of the fusion protein GST‐E‐cadherin was induced by 100 mM IPTG (Beyotime, China). The GST resin (GE Healthcare Biosciences, USA) was equilibrated and then incubated with the supernatants. After removing nonspecific binding, KLF4‐His was expressed in *E. coli* BL21 (DE3) cells and purified using His‐beads. Subsequently, KLF4‐His protein was added and incubated with the resin to allow interaction with the GST fusion protein. Bound protein complexes were eluted and analyzed by SDS‐PAGE and Western blotting to confirm the interaction between KLF4 and E‐cadherin using primary antibodies against His‐tag and GST (Beyotime, China), respectively.

### 5.20 Mouse Models of CRC

Animal experiments were approved by the Animal Ethics Committee of the Affiliated Hospital of Yangzhou University, and conducted in accordance with the US National Institutes of Health “Guidelines for the Care and Use of Laboratory Animals”.

In one experiment, 10 C57BL/6J mice 8–9 weeks old (Shanghai Slack Laboratory Animals, China) were treated for 1 week with streptomycin and penicillin. All mice received an intraperitoneal injection of azoxymethane (AOM; 12 mg/kg), and then exposed to dextran sodium sulfate (DSS) in drinking water (final concentration, 2.5% [w/v]) for five consecutive days. Five mice were randomly selected to receive, by oral gavage, *F. nucleatum* in PBS (10^9^ colony‐forming units, 100 µL) every 2 days for 2 weeks. Mice were euthanized and intestinal tumors as well as other tissues were harvested.

In another experiment, BALB/c nude mice (4 weeks old 16–18 g; Shanghai Slack Laboratory Animals, China) were maintained in a specific pathogen‐free facility. The mice received CRC cells that had been infected for 6 h with *F. nucleatum* at a multiplicity of infection of 1000:1 prior to subsequent experiments (six mice per group).

To create a xenograft model, the LoVo cells (2 × 10^6^ per mouse) were injected subcutaneously into the right flank. Tumor volume was determined every week using the formula 0.5 × length × width^2^. After 4 weeks, mice were euthanized and the xenografts were harvested [11]. For antibody intervention, the mice were treated with an anti‐integrin α5 antibody (10 mg/kg) and IgG isotype control via intraperitoneal injection every 2 days for 2 weeks.

An orthotopic model was created as previously described [52]. Briefly, mice were anesthetized, the cecum was exposed via laparotomy. HCT‐116 cells (2 × 10^6^ per mouse) were injected into the cecal wall. After 30 days, mice were euthanized and intestinal tissues were harvested.

To create a model of liver metastasis, mice were anesthetized, the spleen was exposed via incision, and HCT‐116 cells (1 × 10^6^ per mouse) were injected into the spleen parenchyma. After 2 weeks, mice were euthanized and liver tissues were harvested.

### 5.21 Patient‐Derived Organoids

Fresh colorectal cancer tissues from a colon cancer patient (male, 63 years old, stage III) were collected into centrifuge tubes containing primocin (STEMCELL Technologies, Canada) and diluted in PBS. The tissues were cut into pieces, centrifuged, suspended using cell dissociation reagent (STEMCELL Technologies), and precipitated. The dissociated cells were filtered by a 70‐µm cell sieve, and the filtrate was collected and centrifuged. The pellet was re‐suspended in Matrigel (Corning, USA) and transferred into 24‐well plates, and incubated for 10 min at 37°C with human organoid medium (STEMCELL Technologies) and primocin. The culture medium was replaced every 2 days. During organoids passaging, the organoids were mechanically dispersed with a 1‐mL pippette tip to make them as fragmented as possible and washed with pre‐cooled PBS. The dispersed cells were infected with *F. nucleatum* at a multiplicity of 1000:1.

### 5.22 Statistical Analysis

Data were analyzed using GraphPad Prism 6 (GraphPad, San Diego, CA, USA) or IBM SPSS 20.0 (IBM, Armonk, NY, USA). Differences between two groups were assessed for significance using the unpaired or paired two‐tailed Student's *t*‐test or Mann–Whitney *U* test. Differences among three or more groups were assessed using one‐way ANOVA, followed by Tukey's or Games–Howell post hoc comparisons. Correlation analysis was conducted using linear regression, while the correlation of *F. nucleatum* abundance or expressions of molecules with clinical features were analyzed using Pearson's *χ* ^2^ test or Fisher's exact test. Results were considered significant if *p* was < 0.05.

## Author Contributions

J.L. conceived and designed the experiments. X.Y., X.Q., J.W., and L.L. mainly conducted this study. W.W., J.M., and D.L. collected tissues and data from patients. X.Y. and X.Q. drafted the original manuscript. Y.W., Q.W., and J.L. provided manuscript revision. All authors reviewed and approved the final manuscript.

## Ethics Statement

The clinical studies and animal experiments were approved by the ethics committee of Affiliated Hospital of Yangzhou University (No. 2020‐YKL08‐02 and No. 2020‐YKL04‐Y011). The written informed consent for the use of patient information in medical research was obtained from the patients or their legal guardians.

## Conflicts of Interest

The authors declare no conflicts of interest.

## Supporting information



Supporting Inforamtion

## Data Availability

None.
