# Supplementary material for: Fusobacterium nucleatum Promotes the Growth and Metastasis of Colorectal Cancer by Activating E‐Cadherin/Krüppel‐Like Factor 4/Integrin α5 Signaling in a Calcium‐Dependent Manner
Source: MedComm (2020). 2025 Mar 10;6(3):e70137. doi: 10.1002/mco2.70137 (PMC11892016; doi:10.1002/mco2.70137)
Supplement: Supplementary file 1 — Supporting Inforamtion [file MCO2-6-e70137-s001.pdf]

Fusobacterium nucleatum promotes the growth and metastasis of colorectal cancer by activating E-cadherin/Krüppel-like factor 4/integrin α5 signaling in a calcium-dependent manner

Xuebing Yan[1, 2#], Xiao Qu[3#], Jiaxin Wang[1#], Ling Lu[4#], Wenjuan Wu[5], Jingxian Mao[1], Donglin Li[1], Ying Wang[1], Qing Wei[6], Jianqiang Liu[7*]

[1]Department of Oncology, Affiliated Hospital of Yangzhou University, Yangzhou 225012, China

[2]Jiangsu Provincial Innovation and Practice Base for Postdoctor, Suining People's Hospital, Affiliated Hospital of Xuzhou Medical University, Xu Zhou 221000, China

[3]Department of General Surgery, The First Affiliated Hospital of USTC, Hefei 230001, China

[4]Department of Laboratory Diagnostics, Changhai Hospital, Navy Military Medical University, Shanghai 200433, China.

[5]Department of Oncology, Northern Jiangsu People's Hospital affiliated to Yangzhou University, Yangzhou 225012, China

[6]Department of Pathology, Shanghai Tenth People's Hospital, Tongji University, Shanghai 200072, China

[7]Department of Endoscopy, Fudan University Shanghai Cancer Center, Shanghai 200032, China

[#]These authors contributed equally to this manuscript.

Correspondence: Jianqiang Liu, Department of Endoscopy, Fudan University Shanghai Cancer Center, 270 Dong'an Road, Shanghai 200032, China. Email: ljianqiang@fudan.edu.cn. Tel: +86 18116490497. Fax: +86 18116490497.

Supplementary Tables

Supplementary Table S1. Clinicodemographic characteristics of the two cohorts of colorectal cancer patients in this study.

Characteristic	Cohort 1 (n = 34)	Cohort 2 (n = 40)	p
**Age (year)**	68.97 ± 2.50	65.13 ± 4.22	0.460
**Gender**			0.649
**Male**	23 (67.6)	29 (72.5)	
**Female**	11(32.4)	11(27.5)	
**Tumor location**			0.370
**Colon**	16 (47.1)	23 (57.5)	
**Rectum**	18 (52.9)	17 (42.5)	
**Tumor size, cm**			0.607
**< 5**	21 (61.8)	27 (67.5)	
**⩾ 5**	13 (38.2)	13 (32.5)	
**Differentiation**			0.821
**Well or moderate**	30 (88.2)	37 (92.5)	
**Poor**	4 (11.8)	3 (7.5)	
**T stage**			0.039
**Tis – T2**	11 (32.4)	5 (12.5)	
**T3 – T4**	23 (67.6)	35 (87.5)	
**N stage**			0.370
**N0**	18 (52.9)	17 (42.5)	
**N1 – N2**	16 (47.1)	23 (57.5)	
**M stage**			0.459
**Absence**	33 (97.1)	40 (100)	
**Presence**	1 (2.9)	0 (0)	
**TNM stage**			0.014
**I**	10 (29.4)	2 (5.0)	
**II**	8 (23.6)	15 (37.5)	
**III**	15 (44.1)	23 (57.5)	
**IV**	1 (2.9)	0 (0)	
**Fecal occult blood test**			0.123
**Negative**	5 (14.7)	2 (5.0)	
**Positive**	27 (79.4)	30 (75.0)	
**Unknown**	2 (5.9)	8 (20.0)	
**Serum levels of CEA (mg/L) and CA19-9 (kU/L)**			0.160

**CEA < 5.9 and CA199 < 40**	16 (47.1)	21 (52.5)	
**CEA ⩾ 5.9 or CA199 ⩾ 40**	10 (29.4)	10 (25.0)	
**CEA ⩾ 5.9 and CA199 ⩾ 40**	1 (2.9)	6 (15.0)	
**Unknown**	7 (20.6)	3 (7.5)	
**Percent of tumor cells expressing Ki-67**			0.028
**< 50%**	22 (64.7)	23 (57.5)	
**⩾ 50%**	8 (23.5)	17 (42.5)	
**Unknown**	4 (11.8)	0 (0)	

Values are n ± SD or n (%), unless otherwise noted.

CA19-9, Glucoprotein antigen 199; CEA, carcinoembryonic antigen.

Supplementary Table S2. Correlation of *Fusobacterium nucleatum,* integrin α5 and KLF4 expression level with patient clinical characteristics.

Characteristic	*Fn*		p	integrin α5		p	KLF4		p
	(+) (n=20)	(-) (n=20)		(High) (n=20)	(Low) (n=20)		(High) (n=20)	(Low) (n=20)	
**Age (year)**	63.15±14.99	67.10±12.16	0.366	65.30±15.89	64.95±11.32	0.936	65.25±15.43	65.00±11.94	0.955
**Gender**			0.723			0.723			0.723
**Male**	14 (70.0)	15 (75.0)		15 (75.0)	14 (70.0)		14 (70.0)	15 (75.0)	
**Female**	6 (30.0)	5 (25.0)		5 (25.0)	6 (30.0)		6 (30.0)	5 (25.0)	
**Tumor location**			0.337			0.749			0.749
**Colon**	10 (50.0)	13 (65.0)		11 (55.0)	12 (60.0)		11 (55.0)	12 (60.0)	
**Rectum**	10 (50.0)	7 (35.0)		9 (45.0)	8 (40.0)		9 (45.0)	8 (40.0)	
**Tumor size, cm**			0.736			0.091			0.736
**＜5**	13 (65.0)	14 (70.0)		11 (55.0)	16 (80.0)		14 (70.0)	13 (65.0)	
**⩾ 5**	7 (35.0)	6 (30.0)		9 (45.0)	4 (20.0)		6 (30.0)	7 (35.0)	
**Differentiation**			1.000			1.000			1.000
**Well or moderate**	18 (90.0)	19 (95.0)		18 (90.0)	19 (95.0)		19 (95.0)	18 (90.0)	
**Poor**	2 (10.0)	1 (5.0)		2 (10.0)	1 (5.0)		1 (5.0)	2 (10.0)	
**TNM stage**			0.001			0.013			0.013
**I**	0 (0.0)	2 (10.0)		0 (0.0)	2 (10.0)		0 (0.0)	2 (10.0)	
**II**	3 (15.0)	12 (60.0)		4 (20.0)	11 (55.0)		4 (20.0)	11 (55.0)	
**III**	17 (85.0)	6 (30.0)		16 (80.0)	7 (35.0)		16 (80.0)	7 (35.0)	
**Fecal occult blood test**			1.000			0.842			0.138

**Negative**	1 (5.0)	1 (5.0)	1 (5.0)	1 (5.0)	0 (0.0)	2 (10.0)
**Positive**	15 (75.0)	15 (75.0)	14 (70.0)	16 (80.0)	14 (70.0)	16 (80.0)
**Unknown**	4 (20.0)	4 (20.0)	5 (25.0)	3 (15.0)	6 (30.0)	2 (10.0)

Fn, *Fusobacterium nucleatum.* The cut-off values of *ITGA5* mRNA levels and KLF4 protein levels are determined using the median method.

Supplementary Table S3. Primer sequences used in this study.

Target	Primer sequence (5'-3')
1. Primers used in quantitative PCR	
Fusobacterium nucleatum	Forward primer: CAACCATTACTTTAACTCTACCATGTTCA<br>Reverse primer: GTTGACTTTACAGAAGGAGATTATGTAAAAATC<br>FAM-labeled probe GTTGACTTTACAGAAGGAGATTATGTAAAAATC
SLCO2A1	Forward primer: ATCCCCAAAGCACCTGGTTT<br>Reverse primer: AGAGGCCAAGATAGTCCTGGTAA<br>FAM probe CCATCCATGTCCTCATCTC
ITGA1	Forward primer: CAGCCCCACATTTCAAGTCGT<br>Reverse primer: ACCTGTGTCTGTTTAGGACCA
ITGA2	Forward primer: CAACATGAGCCTCGGCTTGA<br>Reverse primer: GGCTGAGTTGCAGGTGAGAA
ITGA3	Forward primer: GTGGCTTCACCCAGAACACTG<br>Reverse primer: GCTGCCTACCTGCATCGTGT
ITGA4	Forward primer: CCCACTGCCAACTGGCTC<br>Reverse primer: CCTGGCTGTCTGGAAAGTGT
ITGA5	Forward primer: GCCTGTGGAGTACAAGTCCTT<br>Reverse primer: AATTCGGGTGAAGTTATCTGTGG
ITGAV	Forward primer: CATCTGTGAGGTCGAAACAGG<br>Reverse primer: TGGAGCATACTCAACAGTCTTTG
ITGA6	Forward primer: CACTAACAGAGTGGCCGTCCTA<br>Reverse primer: CCCCAATGGCATAGTCTTGTG

ITGB1	Forward primer: TTATTGGCCTTGCATTACTGCT<br>Reverse primer: CCACAGTTGTTACGGCACTCT
ITGB2	Forward primer: CAACGTATGCGAGTGCCATTC<br>Reverse primer: TTCACGGGGTTGTTCGACAG
ITGB3	Forward primer: ACCAGTAACCTGCGGATTGG<br>Reverse primer: CTCATTGAAGCGGGTCACCT
ITGB4	Forward primer: AGACGAGATGTTCAGGGACC<br>Reverse primer: GGTCTCCTCTGTGATTTGGAA
ITGB5	Forward primer: GGATTTGGGTCTTTTGTTGATAAGG<br>Reverse primer: AATGCACGGATTGGTCTGGTA
ITGB6	Forward primer: CATGTCCGCCAGACTGAGG<br>Reverse primer: GAGCCCAGCTCCTTTATTGTG
KLF4	Forward primer: CCGGCGGGAAGGGAGAAGACACTG<br>Reverse primer: TCATAGGGGCGGCCGGGAAGCACT
β-actin	Forward primer: GTCCTGTGGCATCCACGAAACT<br>Reverse primer: TACTTGCGCTCAGGAGGAGCAA
GAPDH	Forward primer: CACATGGCCTCCAAGGAGTAA<br>Reverse primer: TGAGGGTCTCTCTCTTCCTCTTGT

2. Sequences used to knock down target genes after infection with recombinant lentivirus	
shRNA-KLF4	GTTCGTGCTGAAGGCGTCG
shRNA-ITGA5	GACTGGCCGTGTGGTTTTA
shRNA-E-cadherin	GATTGCACCGGTCGACAAA
shRNA-Negative control	GAAGCCAGATCCAGCTTCC
pcDNA-KLF4	Forward primer: ATGAGGCAGCCACCTGGCGAG

	Reverse primer:<br>TTAAAAATGCCTCTTCATGTGTAA
pcDNA-E-cadherin	Forward primer:<br>ATGGGCCCTTGGAGCCGCA<br>Reverse primer:<br>CTAGTCGTCCTCGCCGCCTCCGT
3. Primers used to detect the ITGA5 promoter in chromatin immunoprecipitates	
ITGA5 promoter	Forward primer:<br>TCCCTTTGCAAGACTTTC<br>Reverse primer:<br>TTCCTACAAACCTGGAGAATTGTGAGA

Supplementary Table S4. Antibodies used in this study.

Target antigen	Antibody manufacturer, catalog no.	RRID	Purpose
ITGA5	Abcam, ab150361	AB_2631309	WB, IHC and antibody treatment
ITGA2	Abcam, ab133557	AB_2833020	WB
ITGB1	Abcam, ab52971	AB_870695	WB
ITGB3	Abcam, ab119992	AB_10903329	WB
KLF4	R&D Systems, AF3640	AB_2130224	WB, Co-IP, ChIP, IF
p-KLF4	Affinity Biosciences, AF2437	AB_2845450	WB
E-cadherin	Abcam, ab238099	Not available	WB and Co-IP
FAK	Abcam, ab40794	AB_732300	WB
p-FAK	Abcam, ab81298	AB_1640500	WB
PI3K	Abcam, ab32569	AB_777262	WB
p-PI3K	Abcam, ab182651	AB_2756407	WB
Akt1	Abcam, ab238477	-	WB
p-Akt1	Abcam, ab81283	AB_2224551	WB
C/EBPβ	Invitrogen, PA5-143064	-	WB
p-C/EBPβ	Invitrogen, PA5-105516	-	WB
Sp1	Abcam, ab227383	AB_3073501	WB
p-Sp1	Abcam, ab59257	AB_946335	WB
C-Jun	Abcam, ab31419	AB_731605	WB
p-C-Jun	Abcam, ab32385	AB_726900	WB
HDAC1	Abcam, ab53091	AB_2264059	WB
Ki-67	Santa Cruz, sc-23900	AB_627859	IHC
β-actin	Santa Cruz, sc-81178	AB_2223230	WB
GAPDH	Santa Cruz,, sc-137179	AB_2232048	WB
Secondary antibody	Cell Signaling Technology, 7074	AB_2099233	WB and IHC

ChIP, chromatin immunoprecipitation; Co-IP, co-immunoprecipitation; IF, immunofluorescence; IHC, immunohistochemistry; WB, western blotting.

Supplementary Figures

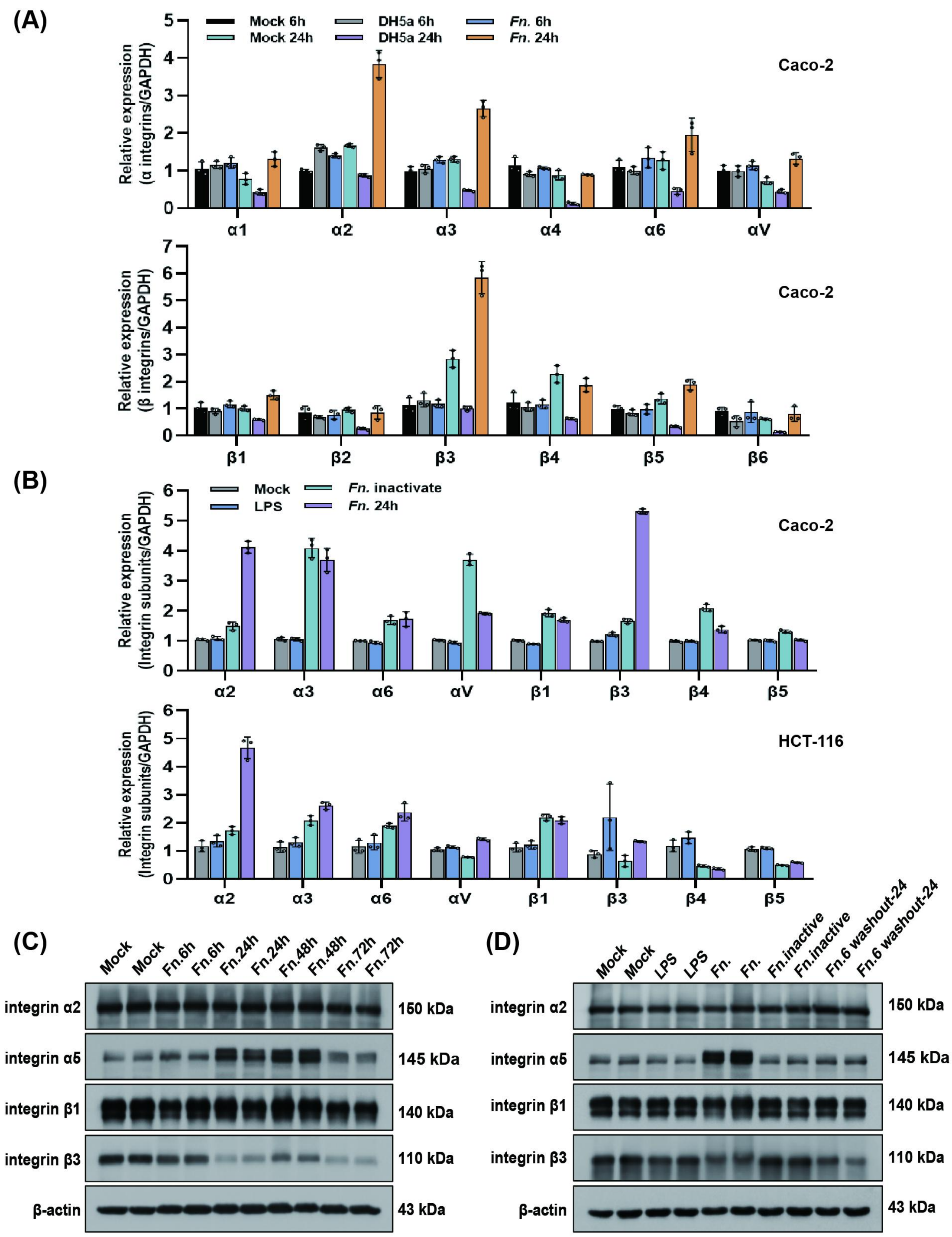

Figure S1. Ability of *Fusobacterium nucleatum* to regulate expression of integrins in colorectal cancer cell lines.

(A) Relative levels of integrin mRNAs in the colorectal cancer (CRC) cell lines Caco-2, normalized to levels of GAPDH mRNA. Cells were infected with bacteria for 6 or 24 h. **(B)** Relative levels of integrin mRNAs, normalized to levels of GAPDH

mRNA, in the CRC cell lines Caco-2 and HCT-116 after treatment with lipopolysaccharide (LPS), mock infection or infection with viable or heat-inactivated *Fusobacterium nucleatum* (*Fn*) for 24 h. **(C)** Western blot of integrins in Caco-2 cells after mock infection or *Fn* infection for 6-72 h. Duplicate samples are shown in all cases. **(D)** Western blot of integrins in Caco-2 cells after 24-h treatment with LPS, infection with viable or heat-inactived *Fn* or treatment with *Fn* followed by exposure to antibiotics for 24 h ("washout"). Error bars on bar plots indicate standard deviation for three independent experiments.

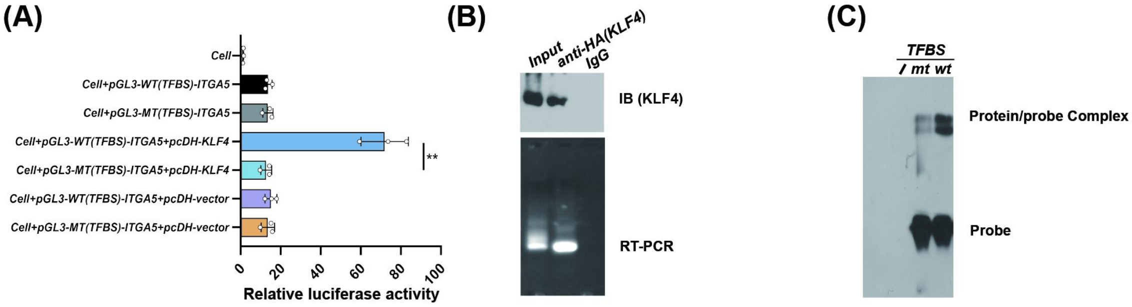

Figure S2. KLF4 activates the transcription of integrin α5.

(A) A luciferase reporter assay was used to detect the transcription activity of an integrin α5 (ITGA5) promoter containing wild-type (WT) or mutated (MT) sequences at the putative binding site of KLF4. 293T cells were co-transfected with plasmids encoding KLF4 and the reporter. **(B)** Chromatin in 293T cells expressing hemagglutinin (HA)-tagged KLF4 was immunoprecipitated using an anti-HA antibody, and the presence of genomic sequences containing the predicted KLF4 binding site in the *ITGA5* promoter was assessed using PCR. **(C)** Electrophoretic mobility shift assay for confirming the binding of KLF4 on the TFBS of *ITGA5* promoter. Error bars on bar plots indicate standard deviation for three independent experiments.**p<0.01.

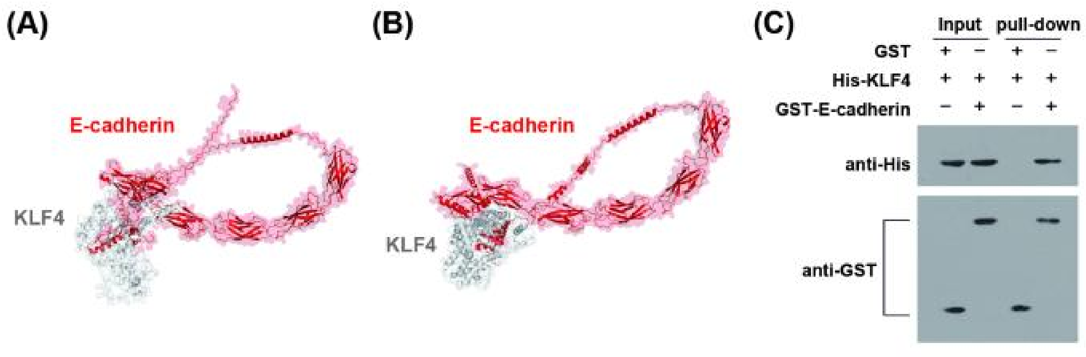

Figure S3. Prediction and validation the direct interaction between E-cadherin and KLF4.

(A-B) Molecular docking models predicting the interaction between E-cadherin and KLF4 (A) or phosphorylated KLF4 (Ser-254) (B). **(C)** The GST pull-down assay confirmed the direct interaction between E-cadherin and KLF4 in *E. coli* BL21 (DE3) cells.

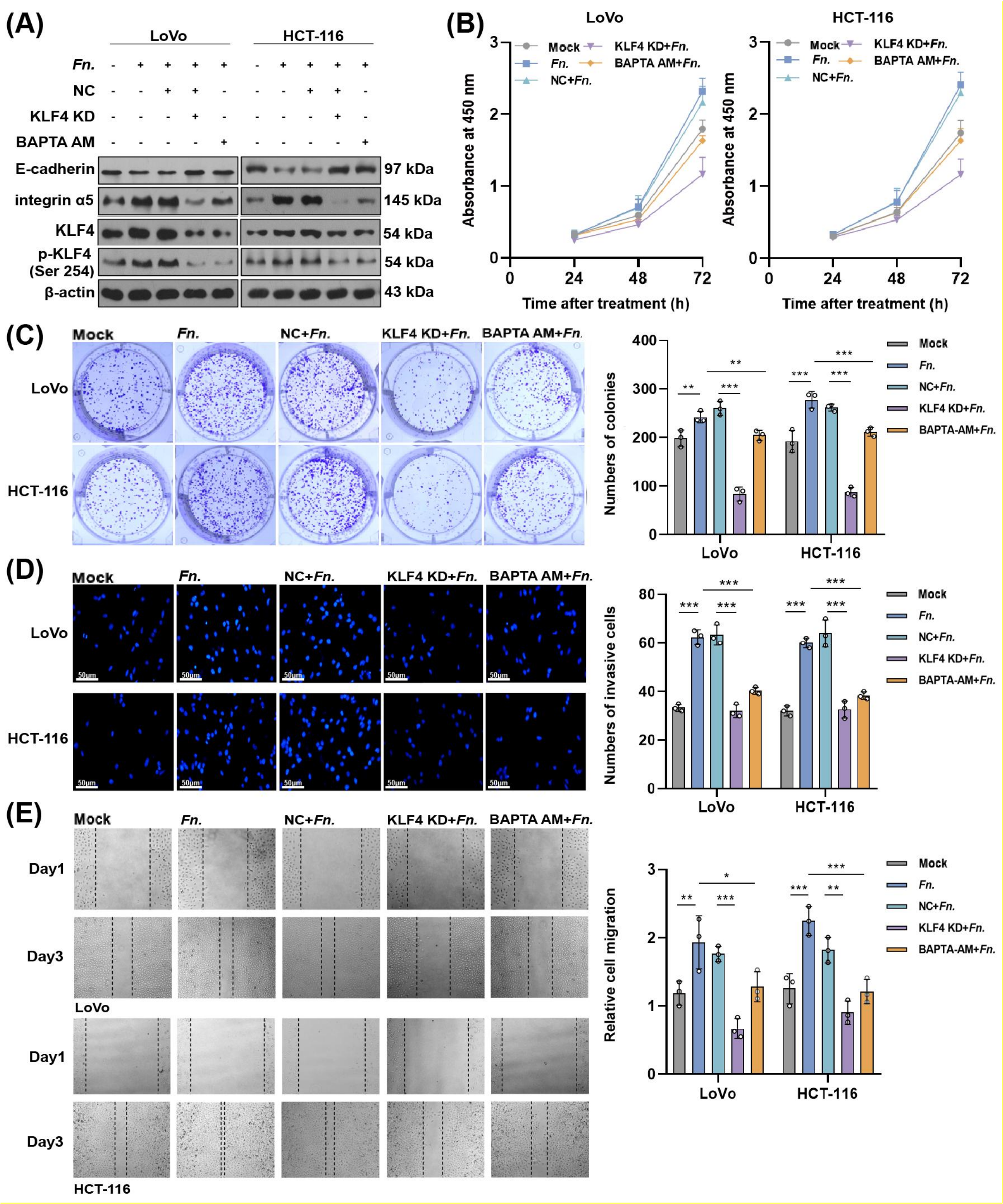

Figure S4. Knockdown of *KLF4* or Ca^{2+} chelation *in vitro* antagonizes the oncogenic effects of *F. nucleatum*.

(A) Western blot to detect the expression of E-cadherin, total and phosphorylated KLF4, and integrin α5 in colorectal cancer (CRC) cells treated with *KLF4* knockdown or BAPTA-AM. **(B)** CCK-8 assay. **(C)** Colony formation assay. **(D)** Transwell assay to detect cell invasion ability. **(E)** Wound healing assay to detect migration ability. Error bars on bar plots indicate standard deviation. *$p<0.05$,

**p<0.01, ***p<0.001.

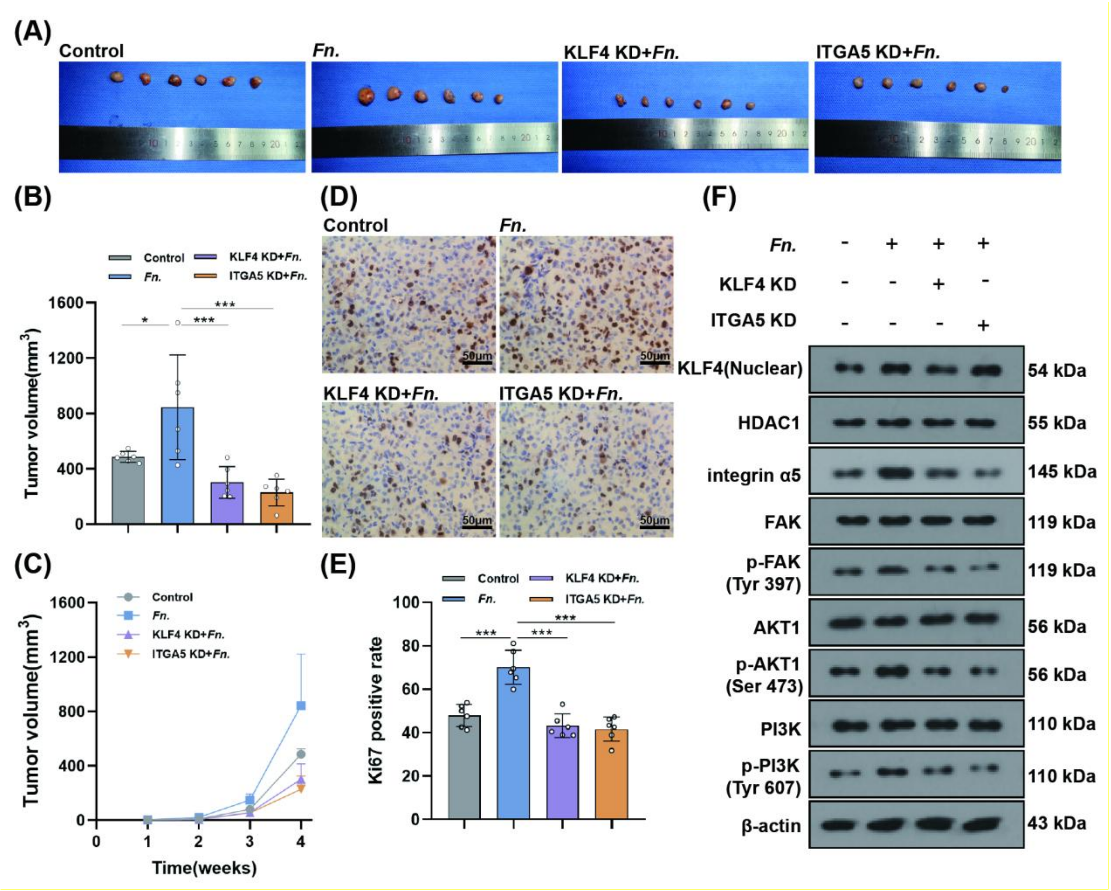

Figure S5. Knockdown of *KLF4* or *ITGA5* antagonizes the oncogenic effects of *F. nucleatum* in colorectal cancer xenografts in nude mice.

(A-B) Photographs of harvested xenografts and final xenograft volumes (6 animals per group). **(C)** Growth of the xenografts over time (6 animals per group). **(D-E)** Representative micrographs of final xenografts after immunostaining against Ki67 and quantitation of Ki67 expression levels (6 animals per group). **(F)** Western blot of total lysates from final xenografts to detect nuclear KLF4, integrin α5 and the phosphorylated forms of FAK, Akt1 and PI3K. Error bars on bar plots indicate standard deviation from six samples per group. *p<0.05, ***p<0.001.

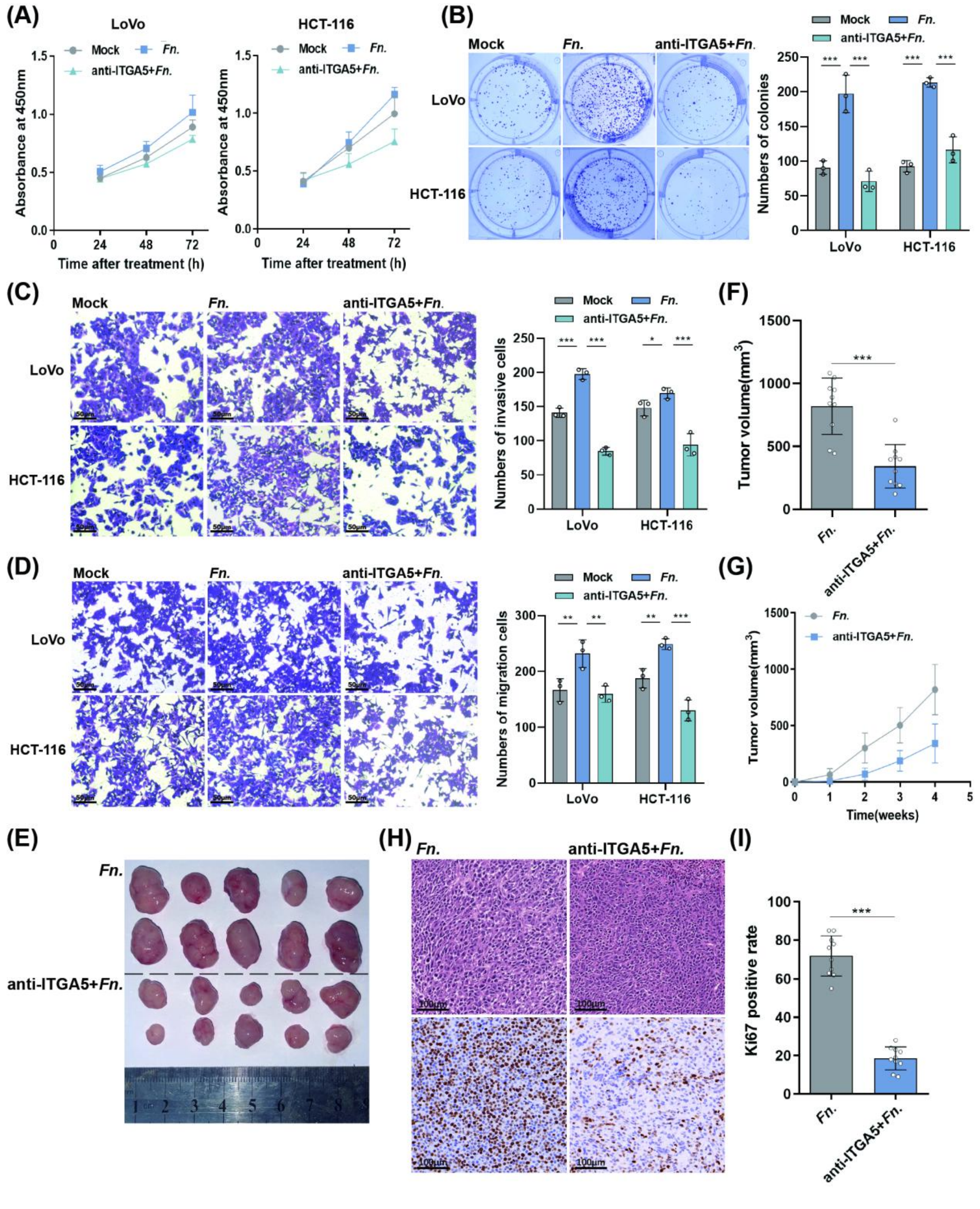

Figure S6. Integrin α5 antibody antagonizes the oncogenic effects of *F. nucleatum in vitro* and *in vivo*.

(A-D) *F. nucleatum* infection promotes the proliferation (A), colony formation (B), invasion (C) and migration (D) of colorectal cancer (CRC) cells *in vitro*, while integrin α5 treatment effectively antagonizes its promoting role. **(E-G)** In the mice carrying subcutaneous HCT-116 xenografts, integrin α5 antibody treatment

significantly slows down the growth speed and decreases the final volumes of *F. nucleatum* infected tumors. **(H-I)** Immunohistochemistry assay demonstrates integrin $\alpha5$ antibody treatment effectively decreases the positive rate of Ki67 in *F. nucleatum* infected xenografts. Error bars on bar plots indicate standard deviation from three or ten samples per group. *$p<0.05$, **$p<0.01$, ***$p<0.001$.
